# Removing Clinical Motion Artifacts During Ventilation Monitoring With Electrical Impedance Tomography: Introduction of Methodology and Validation With Simulation and Patient Data

**DOI:** 10.3389/fmed.2022.817590

**Published:** 2022-01-31

**Authors:** Lin Yang, Shuoyao Qu, Yanwei Zhang, Ge Zhang, Hang Wang, Bin Yang, Canhua Xu, Meng Dai, Xinsheng Cao

**Affiliations:** ^1^Department of Aerospace Medicine, Fourth Military Medical University, Xi'an, China; ^2^Department of Pulmonary and Critical Care Medicine, Xijing Hospital, Fourth Military Medical University, Xi'an, China; ^3^Department of Medical Imaging, Bethune International Peace Hospital, Shijiazhuang, China; ^4^Department of Medical Imaging, Henan Provincial People's Hospital and the People's Hospital of Zhengzhou University, Zhengzhou, China; ^5^Department of Biomedical Engineering, Fourth Military Medical University, Xi'an, China

**Keywords:** thoracic electrical impedance tomography, discrete wavelet transform, motion artifacts, chronic obstructive pulmonary disease, acute respiratory distress syndrome

## Abstract

**Objective:**

Electrical impedance tomography (EIT) is a bedside tool for lung ventilation and perfusion assessment. However, the ability for long-term monitoring diminished due to interferences from clinical interventions and motion artifacts. The purpose of this study is to investigate the feasibility of the discrete wavelet transform (DWT) to detect and remove the common types of motion artifacts in thoracic EIT.

**Methods:**

Baseline drifting, step-like and spike-like interferences were simulated to mimic three common types of motion artifacts. The discrete wavelet decomposition was employed to characterize those motion artifacts in different frequency levels with different wavelet coefficients, and those motion artifacts were then attenuated by suppressing the relevant wavelet coefficients. Further validation was conducted in two patients when motion artifacts were introduced through pulsating mattress and deliberate body movements. The db8 wavelet was used to decompose the contaminated signals into several sublevels.

**Results:**

In the simulation study, it was shown that, after being processed by DWT, the signal consistency improved by 92.98% for baseline drifting, 97.83% for the step-like artifact, and 62.83% for the spike-like artifact; the signal similarity improved by 77.49% for baseline drifting, 73.47% for the step-like artifact, and 2.35% for the spike-like artifact. Results from patient data demonstrated the EIT image errors decreased by 89.24% (baseline drifting), 88.45% (step-like artifact), and 97.80% (spike-like artifact), respectively; the data correlations between EIT images without artifacts and the processed were all > 0.95.

**Conclusion:**

This study found that DWT is a universal and effective tool to detect and remove these motion artifacts.

## Introduction

Electrical impedance tomography (EIT) images the internal impedance distribution from current stimulations and voltage measurements on the body surface (1). As it is a non-invasive and radiation-free imaging modality that can be used in real-time at the bedside, the medical community is very interested in this technique and attempts to apply it into clinical practice, such as monitoring lung ventilation of ICU patients (2), observing the progress of brain injury (3), early detection of breast cancer (4), etc. Among these medical applications, thoracic EIT for ICU patients is one of the most active and promising areas (5), and it focuses explicitly on several directions, including titration of tidal volume or positive end-expiratory pressure, comparison of various ventilation modes, evaluation of lung recruitability and effect of recruitment maneuver, evaluation of suctioning or rehabilitation, monitoring the ventilation distribution for patients with spontaneous breathing, perioperative monitoring and evaluation of regional lung function, etc. (6). In pulmonary and critical care medicine, thoracic EIT is increasingly accepted in hospitals because it can provide different measures of lung function on a regional level and visualize their distribution within the chest, which other established techniques cannot substitute (7).

However, EIT is currently close to but not yet a routine clinical examination method. One of the critical factors limiting its daily use is that EIT might be inherently vulnerable to the source of interference signals, especially to motion artifacts in clinical settings (8). Motion artifacts caused by patients' deliberate movement, medical treatment or nursing are inevitable and may often hinder acquiring, evaluating, and interpreting EIT data (9).

**Figure 1:**
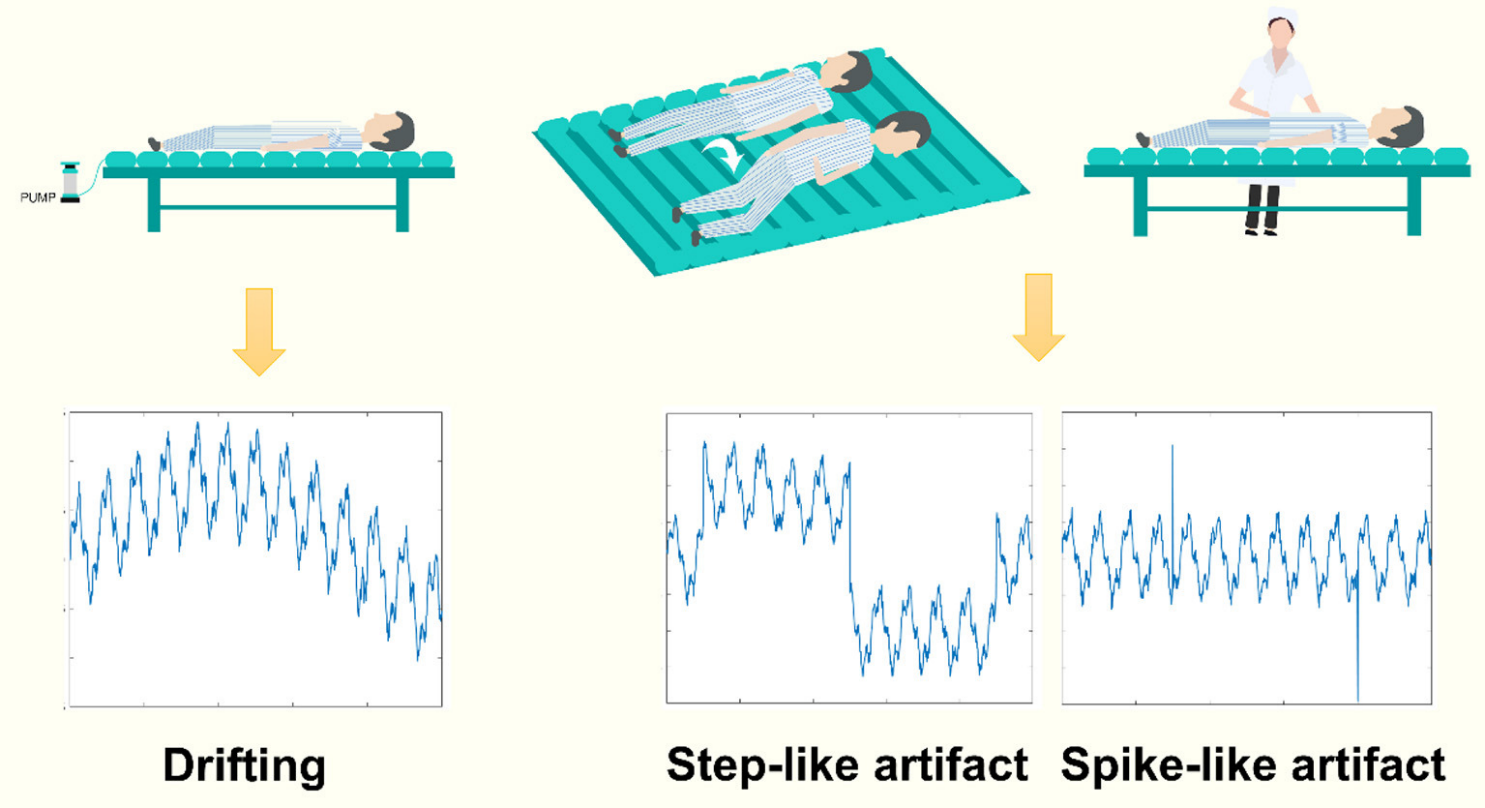
The common types of motion artifacts in clinical EIT. Left: baseline drifting, a slow-varying signal added to the EIT data, often generated by a source of repetitive signals, e.g., an air suspension mattress. Middle and right: the step-like and spike-like artifacts, two types of abrupt varying artifacts, often caused by patients' deliberate body movements or nursing.

Specifically, for thoracic EIT, motion artifacts mainly include three types: baseline drifting, step-like signal, and spike-like signal. The baseline drifting is a slow-varying disturbance, typically generated by repetitive interferences. The pulsating air suspension mattress that repetitively inflates and deflates is a common source of such artifact (9) (Figure 1, left). The step-like signal is a frequent disturbance where the baseline of the impedance signal changes suddenly due to the influence of body movement (e.g., postural change) and does not return to the previous level after the body movement (Figure 1, middle). The spike-like signal is also a frequent disturbance in which the baseline of the impedance signal does return to the previous level after the movement (Figure 1, right). However, to our knowledge, there is currently no study focusing on eliminating motion artifacts in chest EIT for clinical use.

Therefore, we aim to establish a universal signal processing framework for removing these three common types of clinical motion artifacts for chest EIT. The present study introduced the engineering field's discrete wavelet transform (DWT) into our framework and validated it with simulation and patient data.

## Methods

In this section, we introduced the EIT imaging formula and then modeled EIT measured data, which were corrupted by motion artifacts, with DWT; the motion artifacts were subsequently removed by processing the coefficients of DWT. Our framework of motion artifact removal was finally validated with simulation and patient data.

### EIT Imaging Formula

In EIT, 16 electrodes are usually employed to excite the currents and measure the voltages. We used the “adjacent excitation” mode in this study so that for each frame **x**, 16 × 13 = 208 data could be measured. Namely, there are 208 channels of EIT data against time. Next, we can present dynamic and linear EIT image reconstruction by the following formula:


$$\begin{array}{l} \text{y}=\text{B}\left({\text{x}}_{c}-{\text{x}}_{ref}\right) \end{array}$$

where **x**_*c*_ denotes the voltage measurements in the current frame while *x*_*ref*_ represents the measurements of the reference frame. **y** reflects the impedance variation distribution between the current frame and the reference frame. **B** is the inversion of sensitivity matrix (10).

### Modeling Motion Artifacts With DWT

Given that *x*_*measured*_(*n*) is one channel of EIT data affected by motion artifacts, we can represent it as (11–13):


$$\begin{array}{l} x_{measured}\left(n\right)=x_{breathing}\left(n\right)+x_{motion}\left(n\right) \end{array}$$

where *x*_*breathing*_(*n*) is boundary voltage variation from breathings and *x*_*motion*_(*n*) is the motion-artifact component.

**Figure 2:**
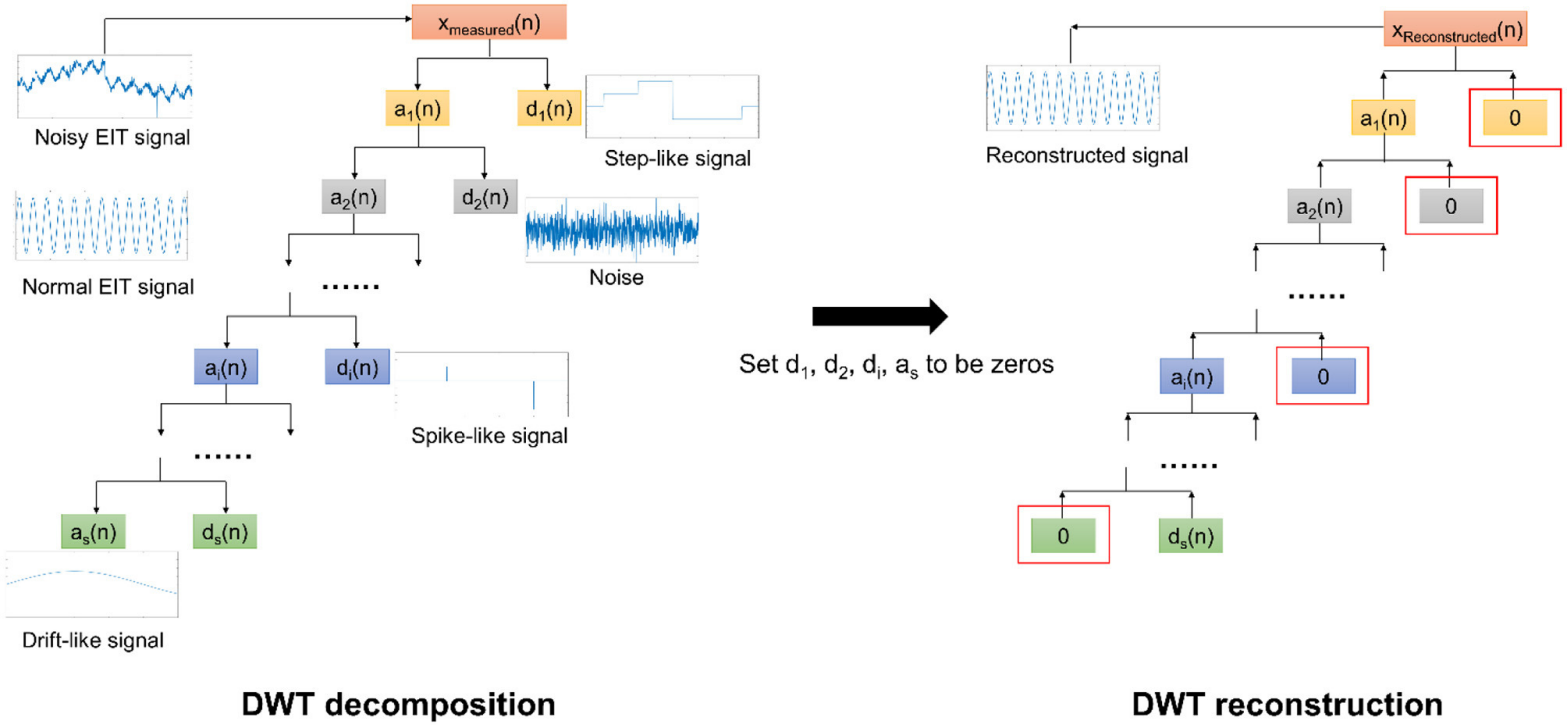
The DWT scheme for EIT motion artifact removal. In the DWT decomposition, the noisy EIT signal is decomposed into a slow-varying signal *a*_1_(*n*)and a fast-varying signal *d*_1_(*n*) at the first level. This processing is repeated with respect to *a*_*i*_(*n*) until those three types of motion artifacts can be portrayed by *a*_*i*_(*n*) and *d*_*i*_(*n*). In the DWT rebuild, we first set these *a*_*i*_(*n*) and *d*_*i*_(*n*) corresponding to motion artifacts to be zeros and then rebuild the EIT signal using the remaining *a*_*i*_(*n*) and *d*_*i*_(*n*). The reconstructed EIT signal would eliminate the motion artifacts.

The DWT processing is shown in Figure 2. It is composed of two parts: decomposition and rebuild (12, 14, 15). In decomposition processing, using the so-called wavelet functions and the scaling functions, DWT decomposes the noisy EIT signal (corrupted by motion artifacts) into a relatively slow-varying signal *a*_1_(*n*) (approximation coefficients) and a fast-varying signal (detail coefficients) at the first step. Theoretically, the obtained signals often represent a type of motion artifact. e.g., *d*_1_(*n*) may reflect the step-like artifacts. Next, we continued to decompose *a*_1_(*n*) to obtain the second-level slow-varying signal *a*_2_(*n*)and the fast-varying signal *d*_2_(*n*). We repeat decomposing the slow-varying signal *a*_*i*_(*n*) at each level until all three types of motion artifacts are correctly portrayed by *a*_*i*_(*n*) or *d*_*i*_(*n*). In addition, as the baseline drifting can be considered a slow-varying signal, it would be depicted by *a*_*s*_(*n*).

Conversely, in the rebuild processing, we constructed the EIT signals with a bunch of *a*_*i*_(*n*) and *d*_*i*_(*n*), also using the wavelet function and the scaling function. Nevertheless, we need to set *a*_*i*_(*n*) and *d*_*i*_(*n*) in relation to the corresponding motion artifacts to be zeros before rebuild. As such, those three types of motion artifacts in EIT signals can be appropriately removed.

In this study, we empirically selected the db8 function as mother wavelet by comprison, and the mathematical scheme is described in detail in the Supplementary Material.

### Simulation Validation

To evaluate the performance of the proposed framework for motion artifact removal, we first carried out experiments using simulated data with Matlab 2016b (MathWorks, Inc., Natick, USA).

We generate a 1-D sine wave signal containing typical oscillations to simulate EIT breathing oscillations with additional Gaussian noise:


$$\begin{array}{l} x_{simulate}\left(t\right)=\frac{1}{2}\overset{2}{\underset{i=1}{\sum }}\mu_{i}\sin\left(\omega_{i}t\right)+\lambda \sigma \left(t\right) \end{array}$$

where ω = 2π*f*, μ represents the oscillation amplitude of the sine wave, σ(*t*) denotes Gaussian white noise, λ represents the amplitude of the Gaussian white noise, and the amplitude range for *x*_*simulate*_(*t*) is from −1 to 1. Here we include two sine waves in the mixed signal: (1) respiratory signal, *f* = 0.25 Hz, μ = 0.9; (2) cardiac signal, *f* = 1 Hz, μ = 0.2. The sampling frequency of the simulated signal was set to 20 Hz and the length was 5,000 samples.

**Figure 3:**
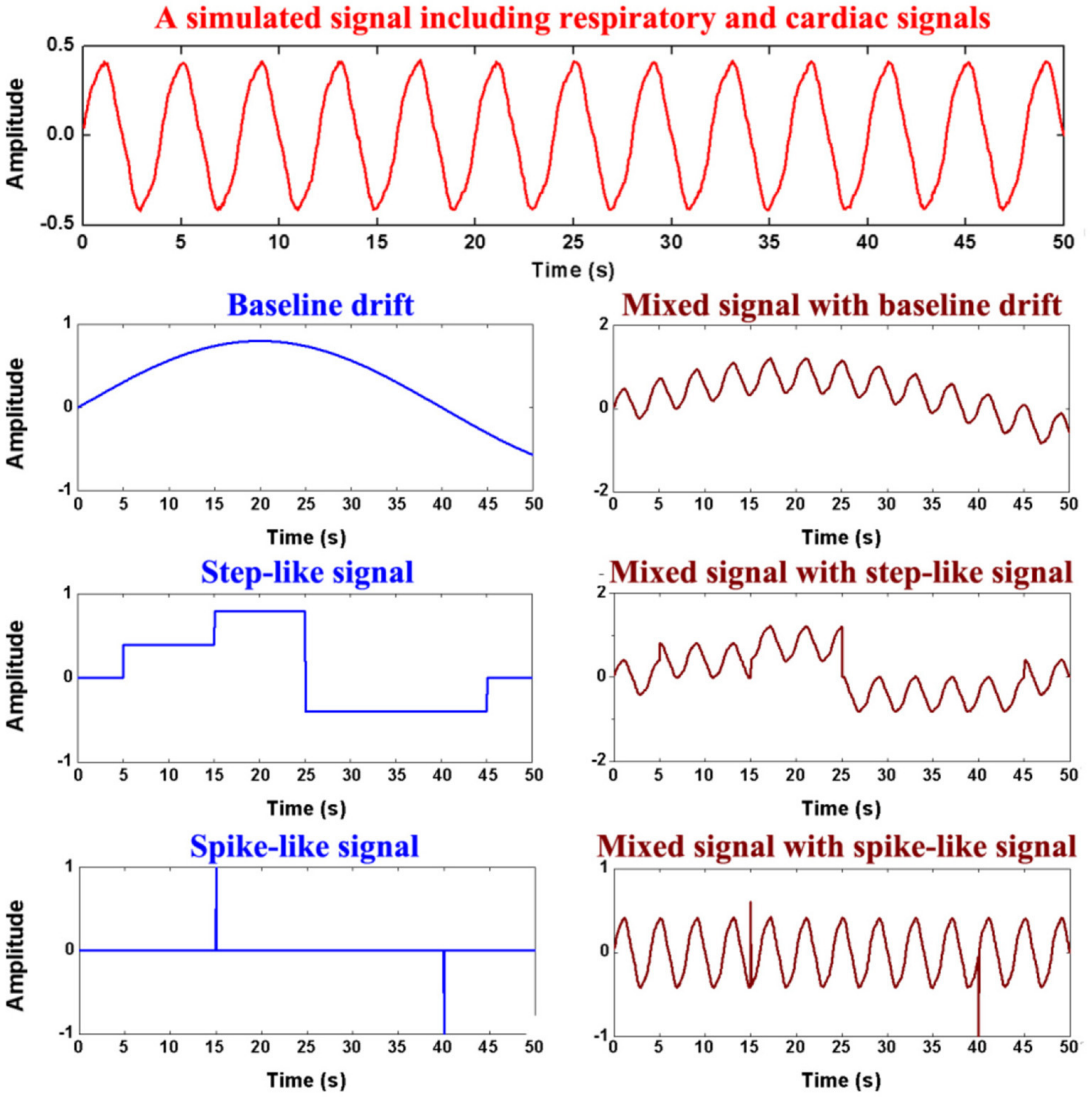
Generation of the simulation data. The first row shows simulated respiratory and cardiac signals, the second row the simulated baseline drifting artifact, the third row the simulated step-like artifact, the last row the simulated spike-like artifact.

Finally, three time-series data were mixed into *x*_*simulate*_, which mimics the three forms of motion artifacts: baseline drifting, step-like signals and spike-like signals (As shown in Figure 3).

After the DWT rebuild, evaluations were carried out to estimate the agreement between the processed signal *y*(*t*) and the original simulated signal without motion artifacts *x*(*t*). The parameters percent root difference (PRD) and coefficient of determination (*R*^2^) were defined as follows (16). PRD evaluates the consistency between *x*(*t*) and *y*(*t*); The greater PRD is, the smaller the consistency is. *R*^2^ evaluates the similarity between *x*(*t*) and *y*(*t*); the larger*R*^2^ means the greater similarity.


$$\begin{array}{l} PRD=100\%\times \sqrt{{\overset{N}{\underset{i=1}{\sum }}{\left(x\left(t_{i}\right)-y\left(t_{i}\right)\right)}^{2}\left(\overset{N}{\underset{i=1}{\sum }}x^{2}\left(t_{i}\right)\right)}^{-1}} \end{array}$$

$$\begin{array}{l} R^{2}=\frac{\overset{N}{\underset{i=1}{\sum }}{\left(y\left(t_{i}\right)-\bar{x\left(t\right)}\right)}^{2}}{\overset{N}{\underset{i=1}{\sum }}{\left(x\left(t_{i}\right)-\bar{x\left(t\right)}\right)}^{2}} \end{array}$$

### Patient Data Validation

The clinical data were acquired in the pulmonary and critical care department of Xijing Hospital, Fourth Military Medical University, Xi'an, China. This study was approved by the human research ethics committee of the Fourth Military Medical University (KY20203282-1), and written informed consent was obtained from patients' nearest relatives. In this scenario, two male patients were included. An EIT electrode belt was attached around the thorax in the fourth intercostal space. The EIT system (PulmoVista 500, Draeger Medical, Luebeck, Germany) was used for recording at a sampling rate of 20 Hz. Image reconstruction was performed using the Graz consensus reconstruction algorithm for EIT (GREIT) algorithm (17).

Patient 1 had moderate acute respiratory distress syndrome. He was mechanically ventilated under volume-control mode. In the EIT data from Patient 1, baseline drifting by a pulsating mattress and the typical step-like artifacts were observed.

Patient 2, with moderate chronic obstructive pulmonary disease, undertook pulmonary rehabilitation. In the EIT data from Patient 2, we found the typical spike-like artifacts caused by his deliberate movements.

After the DWT rebuild, the EIT images before and after being processed were quantitively evaluated for similarity as follows,


$$\begin{array}{l} \text{Imag}{\text{e}}_{error}=\frac{\sum |A_{i}-B_{i}|}{\sum |R_{i}|} \end{array}$$

where A and B represent two different EIT images and *R* denotes the EIT image reconstructed using EIT data with no artifacts. The smaller Image_*error*_ is, the more similar the two images are.


$$\begin{array}{l} \text{Imag}{\text{e}}_{\text{corr}}=\frac{\underset{m}{\sum }\underset{n}{\sum }\left(A_{mn}-Ā\right)\left(B_{mn}-\bar{B}\right)}{\sqrt{\left(\underset{m}{\sum }\underset{n}{\sum }{\left(A_{mn}-Ā\right)}^{2}\right)\left(\underset{m}{\sum }\underset{n}{\sum }{\left(B_{mn}-\bar{B}\right)}^{2}\right)}} \end{array}$$

where A and B represent two different EIT images; *A*_*mn*_ and *B*_*mn*_ denote the pixel values of *m*th row and *n*th column in the image A and B, respectively; Ā and $\bar{B}$ are the mean values of an image A and B, respectively. The Image_corr_ ranges from −1 to 1.1 means the complete positive correlation.

## Results

### Simulation Validation

**Figure 4:**
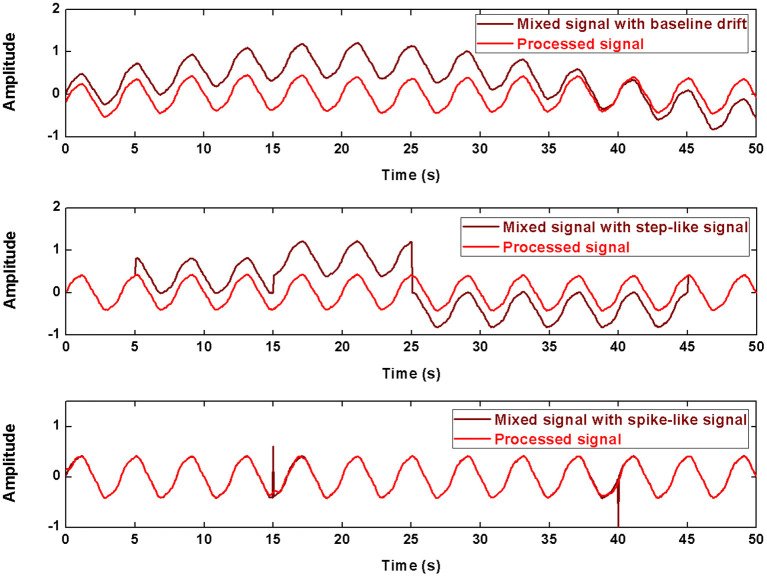
The processed signals in simulation using DWT. The first row shows the result about baseline drifting removal, the second row about step-like artifact removal, the last row about spike-like artifact removal.

The processing results for the simulated data are shown in Figure 4 and Table 1. In baseline detrending, approximation coefficients of Level 6 were excluded in the signal rebuild. In step-like signal correction, only detail coefficients of Level 1 were used to identify the exact location of abrupt changes. Finally, the detail coefficients of Level 1 to the Level 4 were included to perform spike removal. As shown in Figure 4, all three types of simulated artifacts were effectively restrained after being processed. The proposed framework yielded an improvement in signal quality in all three cases. Specifically, it can be demonstrated that the data consistency evaluated by PRD for the drifting removal improved by 92.98%, for the step-like artifact by 97.83%, and for the spike-like artifact by 62.86%; the data similarity assessed by *R*^2^ for the drifting removal improved by 77.49%, for the step-like artifact by 73.47%, and for the spike-like artifact by 2.35%.

**Table 1:** The evaluation parameters before and after being processed in the simulation.

	**PRD**	***R* ^2^**
**Drifting**	1.8653	4.3202
**Drifting-processed**	0.1310	0.9726
**Step-like artifact**	1.6701	3.7737
**Step-processed**	0.0362	1.0011
**Spike-like artifact**	0.1578	1.0151
**Spike-processed**	0.0586	0.9913

### Patient Data Validation

**Figure 5:**
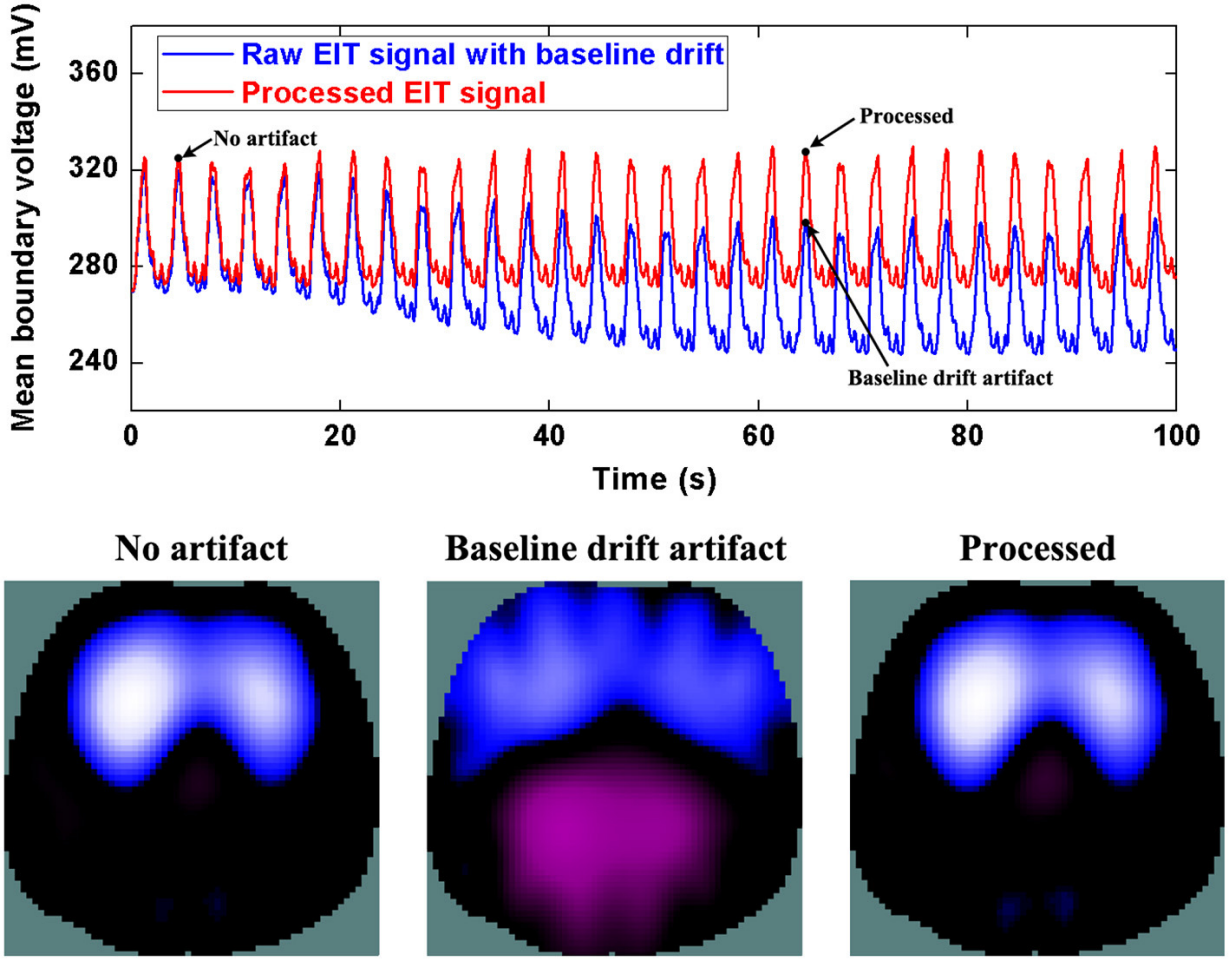
The DWT processing results for baseline drifting removal in patient data. The first row shows the DWT processing result in EIT measured data. The blue line represents the drifting artifact in typical patient data, the red line the corrected data without drifting. The second row includes EIT images without the artifact, with the artifact, and after being processed.

Figure 5 shows the processed results of baseline drifting removal. We can observe the significant artifacts caused by baseline drifting from the boundary voltages and EIT images. After being processed, the artifacts in the boundary voltages and EIT images are notably reduced. In addition, due to the influence of baseline drifting (the purple region in EIT images), we cannot identify whether the ARDS patient's regional lung ventilation distribution only includes the ventral side, i.e., whether the patient's lungs are only ventilated on the ventral side. Nevertheless, we can confirm this after EIT data was processed.

**Figure 6:**
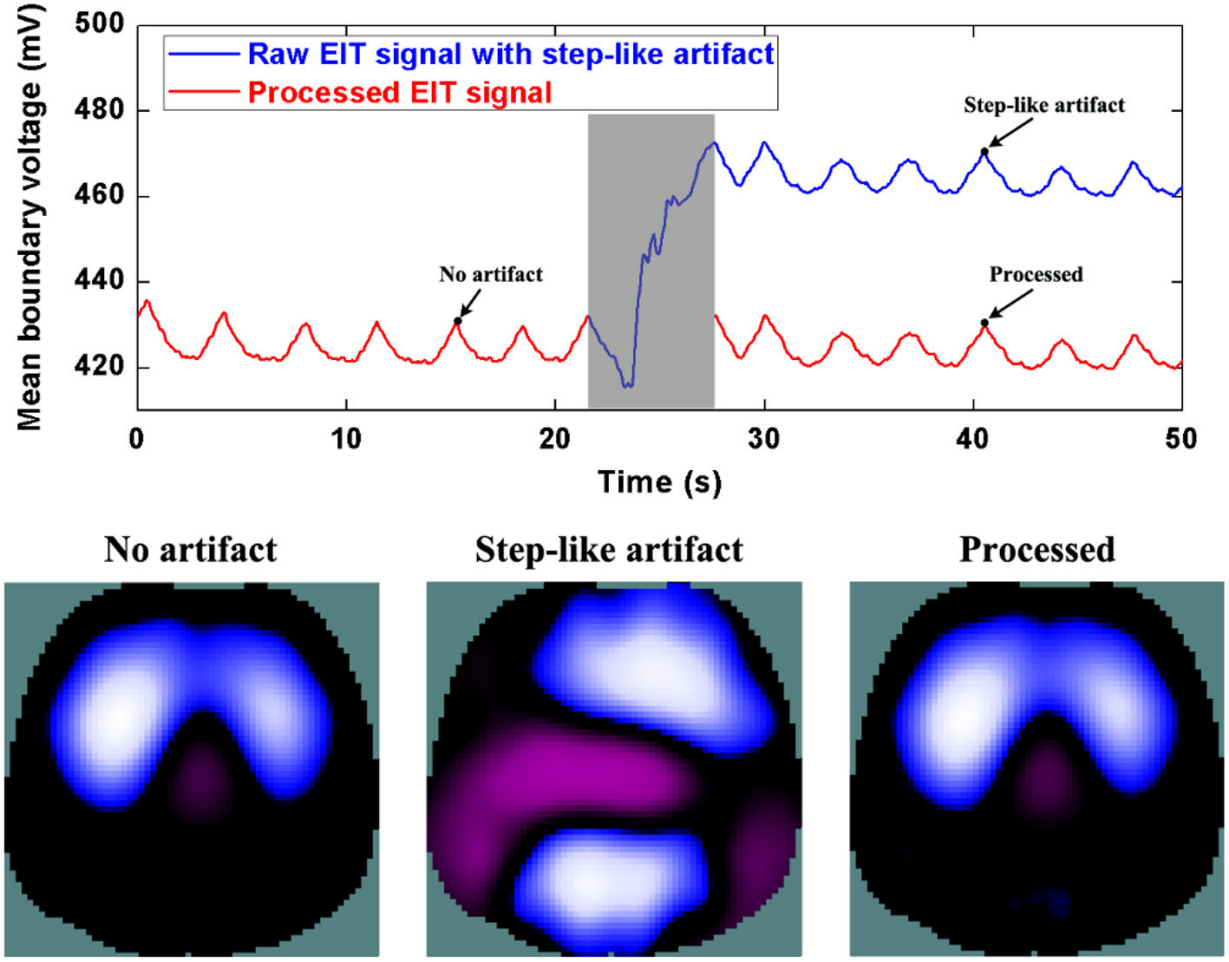
The DWT processing results for step-like artifact removal in patient data. The first row shows the DWT processing result in EIT measured data. The blue line represents the step-like artifact in typical patient data, the red line the corrected data without the step-like artifact. The second row includes EIT images without the artifact, with the artifact, and after being processed.

**Figure 7:**
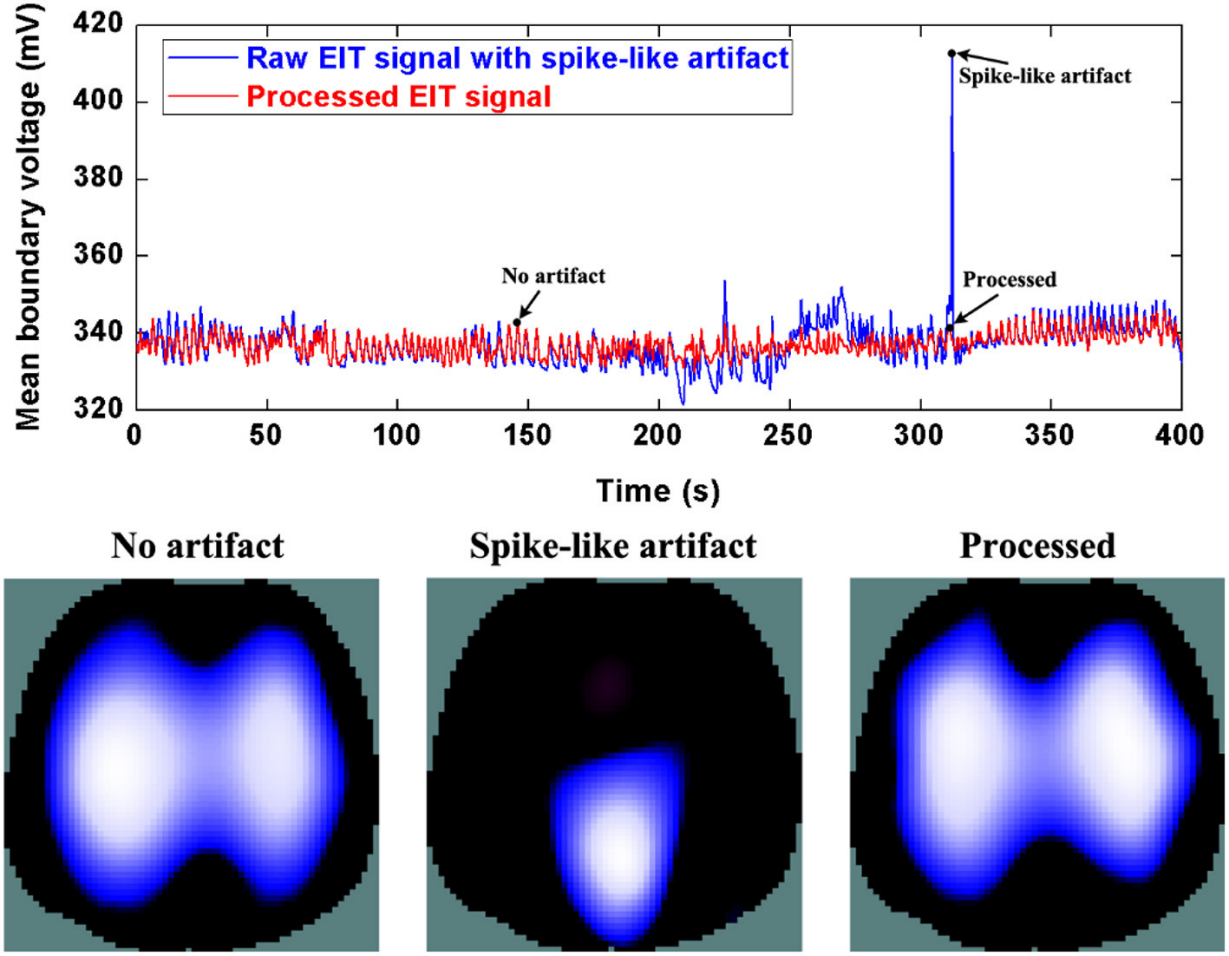
The DWT processing results for spike-like artifact removal in patient data. The first row shows the DWT processing result in EIT measured data. The blue line represents the spike-like artifact in typical patient data, the red line the corrected data without the spike-like artifact. The second row includes EIT images without the artifact, with the artifact, and after being processed.

Similarly, Figure 6 and 7 show step- and spike-like artifact removal processing, with improvement in the averaged waveform of all channels of EIT data and reconstructed EIT images, respectively. As expected, both measurements and images were considerably improved by restoring intrinsic ventilation distribution.

The evaluation parameters for EIT images before and after being processed are listed in Table 2. The image errors decreased by 89.24% (baseline drifting), 88.45% (Step-like artifacts), and 97.80% (Spike-like artifacts). After being processed, the correlations between EIT images without artifacts and the processed ones were all > 0.95.

**Table 2:** The evaluation parameters before and after being processed in the patient data validation.

		**Error**	**Correlation**
**Baseline drifting**	Data w. vs. w/o artifacts	1.58	0.45
	Data w/o artifact vs. DWT	0.17	0.99
**Step-like artifacts**	Data w. vs. w/o artifacts	0.94	0.23
	Data w/o artifact vs. DWT	0.10	0.99
**Spike-like artifacts**	Data w. vs. w/o artifacts	11.57	0.09
	Data w/o artifact vs. DWT	0.25	0.96

## Discussion

Thoracic EIT provides unique information on regional lung ventilation and aeration changes of patients for clinicians at the bedside. However, frequent movement interferences in clinical environments would inevitably compromise EIT data and consequently affect the assessment and interpretation of pulmonary physiological or pathological status. In this study, for three common types of motion artifacts in chest EIT, we, for the first time, proposed to utilize the DWT as a universal framework to remove them. The method was evaluated with both simulated and real patient data. Reduction of ~90% errors in most of the tested scenarios suggested that the proposed method would be potentially applicable and helpful in clinical practice.

The underlying reason for the DWT having a promise to detect and remove these body movement interferences in thoracic EIT is that the motion artifacts may have distinct features in amplitude and duration from the normal ventilation EIT signal. This difference is further highlighted in the wavelet domain due to the inherent localization property of the DWT. When performing DWT to decompose a specific channel of EIT signal into sublevels, a segment of approximation wavelet coefficients (corresponding to lower frequency signals) and several segments of detail wavelet coefficients (corresponding to higher frequency signals) were finally obtained. Furthermore, it was shown that those three types of motion artifacts were on the different sublevels. Namely, those motion artifacts could be portrayed and rebuilt using the related sublevels of wavelet coefficients. Therefore, we may attenuate them efficiently by suppressing the corresponding wavelet coefficients or abandoning them after detecting them. In fact, due to DWT's inherent advantages, it had not only been suggested for removing the motion artifacts for brain EIT (18) but also for other biosignals like functional near-infrared spectroscopy (11), cardiac electrophysiology (19), magnetocardiography (14), etc.

Theoretically, those motion interferences may change the external pressure exerted on the EIT electrodes and affect both the current injection and voltage measurement through these electrodes. It would eventually lead to changes in electrode-skin contact impedance. Currently, there are two directions of methods addressing this issue.

Several researchers attempted to improve EIT imaging algorithms using complete electrode models. Their ideas are to separate contact impedance changes from image reconstruction and reduce their effects on specific elements in the finite element models (20–22). These proposed methods to eliminate contact impedance artifacts might also improve clinical data quality affected by body movements. Also, some researchers may consider updating the EIT reference data when motion artifacts occur. They used synthetic reference data to replace the actual EIT reference state to cancel those artifacts to a certain extent (23).

Another direction to solve the issue is signal processing methods on biosignals, which is the path we chose in this study. The reason for choosing the DWT method in this study is that DWT can portray three different body motion artifact signals simultaneously in different wavelet domains; by one discrete wavelet transform, the characteristics of three types of motion artifact signals can be presented simultaneously; furthermore, by attenuating these wavelet coefficients that respond to those artifacts and reconstructing the original signal, the effects of those artifact signals can be reduced simultaneously. In short, one DWT can filter out three kinds of motion artifacts at the same time. Besides the wavelet-based approaches, Wiener filtering has also been suggested for removing motion artifacts in fNIRS if prior knowledge of the original signal's power spectrum is known (24). Kalman filtering has also been applied to fNIRS and photoplethysmography with a prior assumption on the distribution of noise that models the artifacts (25). Scholkmann et al. also proposed an *ad hoc* algorithm in fNIRS, in which the moving standard deviation scheme was used to detect motion artifacts and spline interpolation was used to model and correct them (16). Specifically, the signal processing method based on sparse and redundancy representation, e.g., robust PCA, might be a new direction to address the issue if we can model the measured data as a low-rank matrix (26).

This study has several limitations. First, real-time processing by DWT was not considered, and all the analyses were performed offline. The reason is that the purpose of this preliminary study mainly focused on the feasibility of DWT to detect and remove the motion artifacts. From the present results, we would continue this work in an attempt to perform DWT in real-time. Second, in this study, we dealt with only one type of movement artifact at a time. Therefore, it is necessary to confirm whether the DWT method could simultaneously detect and attenuate or remove three types of movement artifacts. Third, except for the pulsating mattress, the sources of interference from other nursing and monitoring devices were not considered, e.g., impedance pneumography, continuous cardiac output monitor. Therefore, we need to determine whether these interferences could be reduced or removed using the DWT in future studies.

## Conclusion

Thoracic EIT in clinical practices may often be disturbed by different body movements. The typical artifacts caused by body movements include baseline drifting (by a pulsating mattress), step-like and spike-like impedance signals. This study found that DWT is a universal and effective tool to detect and remove or attenuate these motion artifacts. In future studies, simultaneous processing of those three types of motion artifacts in real-time needs further consideration.

## Data Availability Statement

The original contributions presented in the study are included in the article/Supplementary Material, further inquiries can be directed to the corresponding authors.

## Ethics Statement

The studies involving human participants were reviewed and approved by the Human Research Ethics Committee of Xijing Hosptical, The Fourth Military Medical University. The patients/participants provided their written informed consent to participate in this study.

## Author Contributions

All authors listed have made a substantial, direct, and intellectual contribution to the work and approved it for publication.

## Funding

The work was supported in part by the National Natural Science Foundation of China (61901478, 52077216, 62001421, and 51837011), the Medical Program of AFMU (2018HKTS10, 2019ZTC01), the Equipment Program of PLA (KJ2018-2019C132), Xijing Hospital Promoting Project (XJZT21CM13) and the Everest Program of AFMU (2019ZFB002).

## Conflict of Interest

The authors declare that the research was conducted in the absence of any commercial or financial relationships that could be construed as a potential conflict of interest.

## Publisher's Note

All claims expressed in this article are solely those of the authors and do not necessarily represent those of their affiliated organizations, or those of the publisher, the editors and the reviewers. Any product that may be evaluated in this article, or claim that may be made by its manufacturer, is not guaranteed or endorsed by the publisher.
