# Supplementary material for: Removing Clinical Motion Artifacts During Ventilation Monitoring With Electrical Impedance Tomography: Introduction of Methodology and Validation With Simulation and Patient Data
Source: Front Med (Lausanne). 2022 Jan 31;9:817590. doi: 10.3389/fmed.2022.817590 (PMC8841770; doi:10.3389/fmed.2022.817590)
Supplement: Supplementary file 1 [file Data_Sheet_1.PDF]

Supplementary Material

1 Supplementary Data

Supplementary Material should be uploaded separately on submission. Please include any supplementary data, figures and/or tables. All supplementary files are deposited to FigShare for permanent storage and receive a DOI. Each channel of the observed contaminated signal $s_{mix}(\mathrm{t})$ is composed of the desired signal plus an interference term:

$$s_{mix}(t) = s_{resp}(t) + s_{contam}(t)$$

where $s_{resp}(t)$ represents boundary voltage variation from respiration and $s_{contam}(t)$ is the motion-artifact components. We then use the wavelet transform method to carry out the decomposition, in which the signal $s_{mix}(t)$ can be expanded using DWT as

$$s_{mix}(t) = \sum_{k} v_{j_0 k}\phi_{j_0 k}(t) + \sum_{j=j_0}\sum_{k} w_{jk}\psi_{jk}(t)$$

where $\phi_{jk}(t) = 2^{j/2}\phi(2^j t - k)$ denotes the scaling function for reconstruction, $\psi_{jk}(t) = 2^{j/2}\psi(2^j t - k)$ represents the corresponding wavelet function. j and k are dilation and translation parameters, respectively. v represents the approximation coefficients and w_j represents the detail coefficients at the j th level. The DWT of the observed signal is as follows:

$$w_{jk} = \sum_{l} g(l-2k)v_{j+1}(l)$$

$$v_{jk} = \sum_{l} h(l-2k)v_{j+1}(l)$$

where $j = j_0, \ldots, J-1$, k indicates the k th element in the coefficients vector. $g(l-2k)$ and $h(l-2k)$ are high- and low-pass filters of the wavelet filter bank, respectively. The coefficients can be expanded as

$$w_{jk} = w_{jk,s_{resp}} + w_{jk,s_{contam}}$$

$$v_{jk} = v_{jk,s_{resp}} + v_{jk,s_{contam}}$$

We can organize the decomposition coefficients into the form of a matrix as:

$$\mathbf{W} = [\mathbf{W}_{J-1} \quad \mathbf{W}_{J-2} \quad \dots \quad \mathbf{W}_0 \quad \mathbf{V}_0]$$

where $\mathbf{W}_j$ are the wavelet coefficients and $\mathbf{V}_0$ are the scaling coefficients. As the three common types of motion artifacts are undesirable, the corresponding wavelet coefficients or scaling coefficients would be reduced.
